# Feasibility of high resolution ultrasound for SLAP tears of the shoulder compared to MR arthrogram

**DOI:** 10.1259/bjro.20190007

**Published:** 2019-06-12

**Authors:** Akeel Alali, David Li, Sandra Monteiro, Hema Choudur

**Affiliations:** 1 Department of Radiology, McMaster University, Hamilton, Canada; 2 Department of Clinical Epidemiology and Biostatistics, McMaster University, Hamilton, Canada

## Abstract

**Objectives:**

The purpose of this prospective pilot study was to evaluate the feasibility and accuracy of high resolution ultrasound in the detection of superior labral anteroposterior (SLAP) tears of the shoulder compared to MR arthrogram.

**Methods and materials:**

48 adult patients were included in the study. All patients had high resolution ultrasound of the superior labrum and biceps labral anchor prior to MR arthrogram. Ultrasound and MR arthrograms were evaluated separately for the presence or absence of SLAP tear using the same grading. The presence or absence of a tear and grading of the tears on MR arthrograms and ultrasound were compared and evaluated using κ statistics.

**Results:**

Both MRI and ultrasound demonstrated a SLAP tear in 27 of the 48 patients. MRI and ultrasound were in agreement on the absence of a tear in 19 patients. There was a disagreement between MRI and ultrasound in 2 of the 48 patients regarding the existence of a tear. The two modalities demonstrated substantial agreement on the presence or absence of a tear ( κ = 91.4 %, *p* < 0.001) as well as the grading of the tear ( κ = 84.4 %, *p* < 0.001).

**Conclusions:**

In this pilot study, the feasibility and accuracy of high resolution ultrasound for SLAP tears were evaluated and compared with MR arthrogram. MRI and ultrasound demonstrated substantial agreement on the presence or absence of SLAP tears and grading of the tears.

**Advances in knowledge:**

This pilot study explores and supports the use of ultrasound as a screening tool for SLAP tears, especially as it is readily available, fast and inexpensive.

## Introduction

The superior labrum anteroposterior (SLAP) lesions of the shoulder are common injuries. The SLAP tear itself is the commonest labral pathology in the shoulder accounting for 80–90% of cases and had been described in 6% of shoulder arthroscopies.^1^ Additionally, the number of shoulder cases that necessitated surgical repair has increased during the last few years from 9.4 to 10.1% particularly with the increase in shoulder arthroscopies.^2,3^ The literature revealed significant increase in the number of arthroscopic SLAP repairs during the last few decades.^4^

There are several mechanisms that might result in a SLAP tear which is particularly common in throwing athletes and swimmers where the mechanism of injury is traction on the arm as a result of a sudden pull on the arm or as a result of repetitive overhead motion.^5^ SLAP lesions may also occur after a fall on an outstretched hand or a direct trauma to the shoulder.

Accurate diagnosis and grading of SLAP lesions of the shoulder can be challenging. There are so many different physical examination tests for superior labral tears which have described in the literature and demonstrated variable accuracies but are generally low.^6^ According to the literature, the Compression-Rotation test has the highest accuracy among the other physical tests (sensitivity 0.43, specificity 0.89) which is still low. Diagnostic MR and CT arthrogram play a major role in the diagnosis and grading of SLAP lesions. MR arthrogram is currently considered the best imaging test to evaluate SLAP lesions with sensitivity ranged from 82 to 100% as well as specificity from 71 to 98%.^7^ Although ultrasound is readily available, fast and inexpensive, there are only few prior published papers comparing ultrasound to MR arthrogram. Therein, the purpose of this prospective pilot study was to evaluate the feasibility and accuracy of high resolution ultrasound in the detection of SLAP tears of the shoulder compared to MR arthrogram.

## Methods and materials

This study was approved by Hamilton Integrated Research Ethics Board. The main conception, design and image interpretation for this study was by HC. Data interpretation, analysis and drafting of the article was by AA. DL was responsible for data collection.

Statistical analyses were conducted (by SM) using SPSS v. 25.0 (IBM Corp. Released 2017. IBM SPSS Statistics for Windows, v. 25.0. Armonk, NY: IBM Corp).

48 adult patients were included in this prospective study over 2-year period from Jul 2014 to Dec 2016. The outpatients were referred to the radiology department by several orthopedic surgeons with a clinical suspicion of a SLAP tear. Pediatric patients (below age of 16 years) were excluded from the study. Patients who were with large body habitus where there was suboptimal penetration of the ultrasound beam on ultrasound scanning were also excluded from the study. Large body habitus causing inadequate penetration of the ultrasound beam was a limitation of the ultrasound technique. We have not used any specific parameters in the study design as criteria for cut-off for large body habitus such as BMI, muscle or fat thickness measurements. If the anterior glenohumeral joint was visualized on ultrasound after making appropriate depth changes as one would do with ultrasound of any other body part, we included such patients in our study. If the anterior glenohumeral joint was not appreciated on ultrasound, we did not include such patients in our study as the labrum is seen immediately deep to the joint space, along the superior bony glenoid and with inadequate visualization of the anterior joint, the labrum would be obscured.

All included patients had high resolution ultrasound of the superior labrum and biceps labral anchor prior to MR arthrogram. Using a linear MSK probe, serial images of the superior labrum were obtained by a MSK staff radiologist (HC), with overall 22 years’ experience in ultrasound scanning and 16 years post-musculoskeletal fellowship training in ultrasound scanning and interpretation of MRI/MR arthrograms. As it is a pilot study, the characteristics of a normal labrum and torn labrum were identified based on the standard description of SLAP tears, but were classified into four categories.^8^ Type 1 is fraying of inferior margin; Type 2 is defined as a cleft with fluid at the attachment of the labrum to the bony glenoid; Type 3 is a cleft within the labrum, perpendicular to bony glenoid; Type 4 is a cleft within the labrum perpendicular to the bony glenoid and extending into the biceps labral anchor. This classification for the purpose of the study kept the division of labral tears simple to comprehend and identify separate from one another. It was then compared to the very basic same grading of standard SLAP tears classification on coronal oblique MR arthrogram based on the articular surface fraying and the direction of the slap tear (cleft filled with gadolinium) relative to the attachment (base) of the labrum to the bony glenoid (parallel or perpendicular to the base of the labrum). As the comparison was made to standard MR arthrogram images, for the purpose of the study, we called tears based on the fraying and the presence of cleft at labral attachment of the bony glenoid as Slap 1 and 2 respectively. Type 2 is a tear between the superior glenoid labrum and adjacent bony glenoid. Type 3 is a tear perpendicular to the labral attachment to the bony glenoid. Type 4 is a tear of the superior labrum extending into the biceps labral anchor.

The MR arthrograms (coronal *T*_1_ fat sat sequences) were evaluated separately for a SLAP tear using the same grading as ultrasound. The ultrasound assessment was performed prior to the intraarticular gadolinium injection and MRI exam. The radiologist was also blinded to patient’s identity, clinical scenario and ultrasound findings when assessing the MR arthrograms. The MRI interpretation was performed weeks and in some cases few months after the ultrasound examination. MRI and ultrasound findings for the presence/or absence of a tear and for grading of the tears was assessed with κ statistics, as this is recognized as widely accepted measure of agreement.

The concomitant normal variants were not addressed as part of this study design as these variants could sometimes be difficult to identify and differentiate from a tear on MR arthrogram which was the standard of comparison for the purpose of this study.

### Ultrasound technique

**Figure 1.**
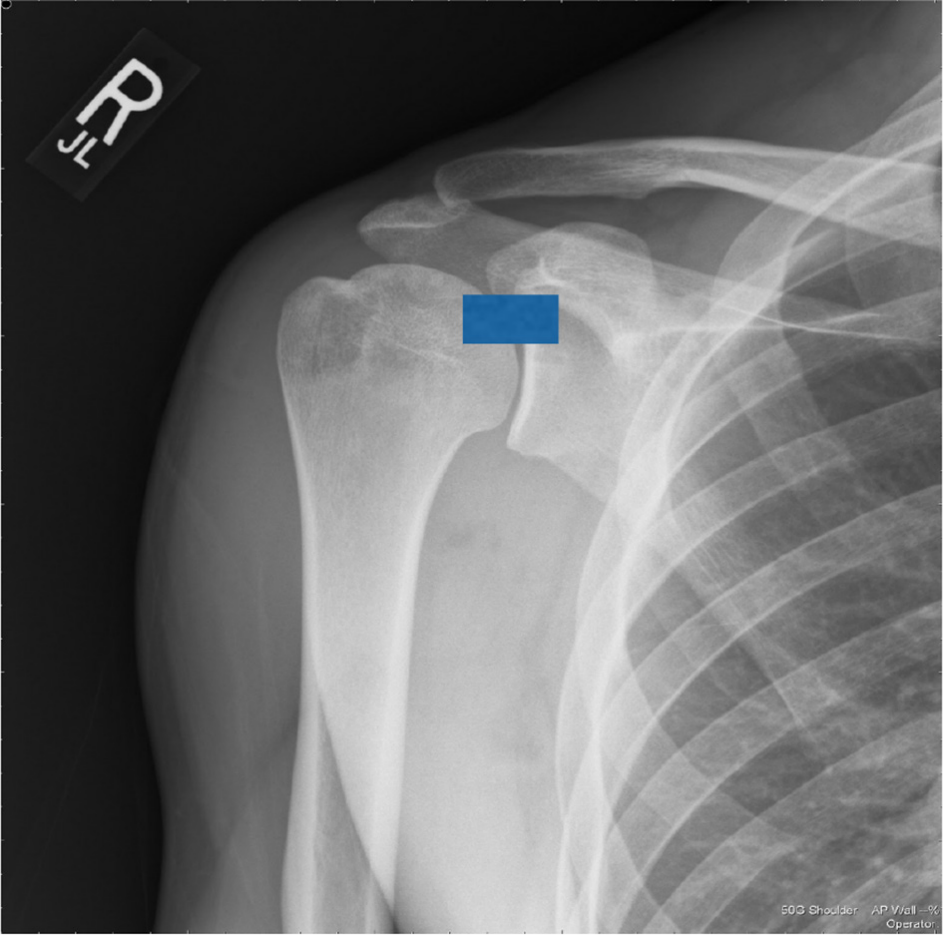
Position of the ultrasound probe at the level of the coracoid and humeral head.

All the cases were scanned by the same GE Logic 9 Ultrasound machine. Using linear high frequency MSK transducer (12 MHz), the long axis of the transducer was placed in the mediolateral plane, at the level of the coracoid (Figure 1). It was moved slightly superiorly and posteriorly in the same plane and the long head of biceps labral anchor was identified. Once the biceps labral anchor was identified, the depth and gain was adjusted till the labrum was clearly demonstrated.

**Figure 2.**
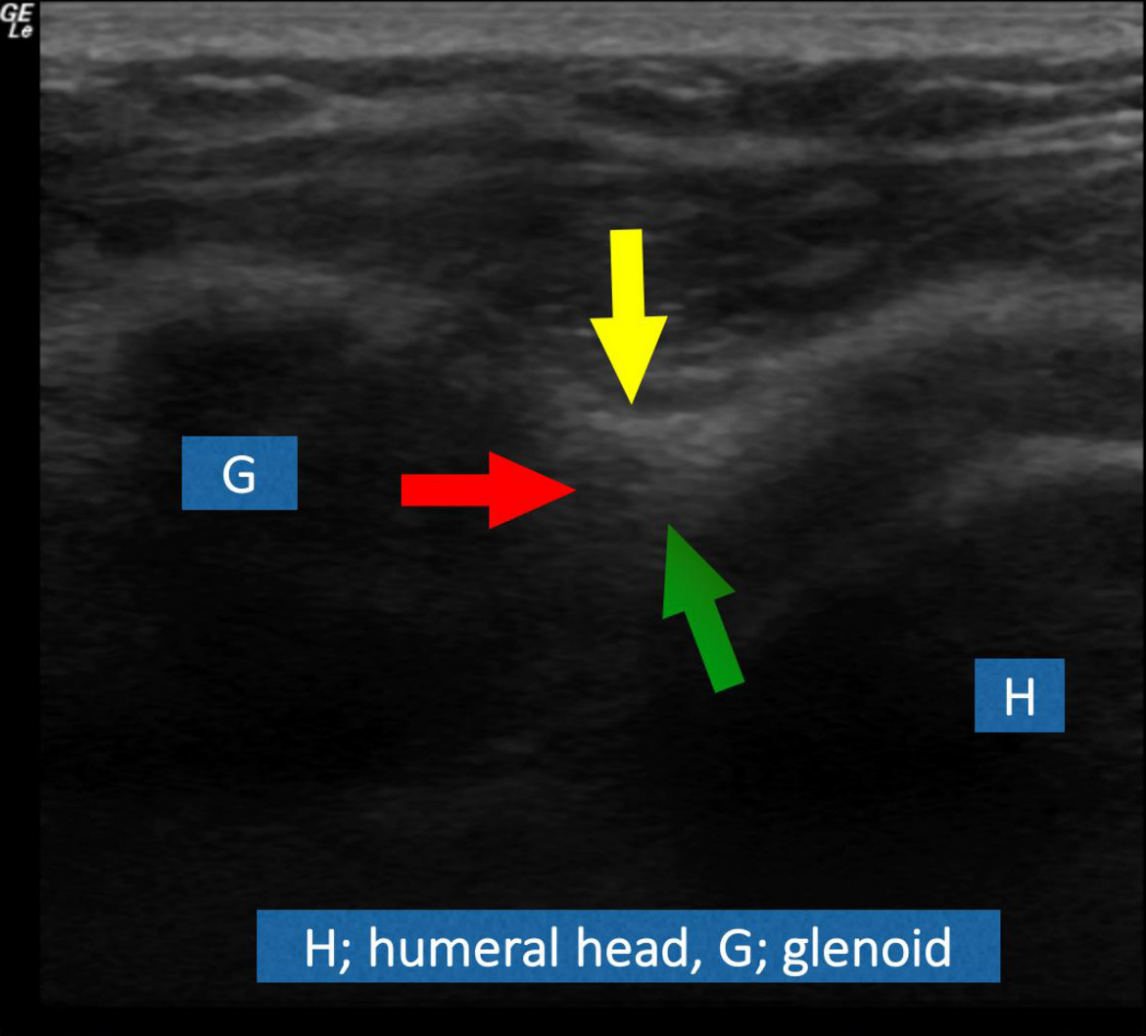
The normal labrum is seen as a triangular echogenic focus as shown by the arrows. Green: articular surface, Red: glenoid surface, Yellow: non-articular surface.

In this plane, the probe was tilted vertically (first anteriorly and then posteriorly, tilt described relative to the transducer surface on the skin), obtaining serial images of the anterior and then posterior aspects of the superior labrum. The biceps anchor was in the middle of this set of images. These images correspond to the labrum as seen on the coronal oblique MR arthrogram. The normal labrum is seen as a triangular echogenic focus as shown by the arrows (Figure 2). All serial images were reviewed and where there was a cleft in the labrum (triangular hyper echogenicity) or fraying of its margins, that particular image was utilized for the study, and labelled as abnormal labrum (from a tear). If there were more than one such abnormal images showing the cleft or fraying, the single best image that demonstrated the abnormality clearly was utilized for the study.

### MRI arthrogram technique

10 cc of diluted gadolinium (2.5 ml of gadolinium in 250 ml sterile saline) was injected into the glenohumeral joint utilizing the standard anterior approach under fluoroscopy. The patient was scanned in a 1.5 T Siemens MRI, using standard shoulder coil to obtain the standard departmental protocol for MR arthrogram. This included the coronal oblique *T*_1_ fat sat sequence which was used for the purpose of the study.

## Results

There were 49 adult patients who were referred by orthopedic surgeons to rule out SLAP tear from Jul 2014 to Dec 2016. These patients had several mechanisms of injury. Recurrent subluxation is the commonest among the other mechanisms (*n* = 16) representing 33.33% of the cases. A lot of these cases had history of trauma for example MVC, sport or work-related injury. One case was excluded as the MRI was terminated due to claustrophobia. 48 cases were valid for analysis with no missing data with regards to the existence of a SLAP tear. 46 cases were valid for analysis for the type of SLAP tear, 2 cases were excluded as the type of SLAP tear was ambiguous and indefinite on both modalities, *i.e*. Type 1–2 and 2–3 (Flow chart).


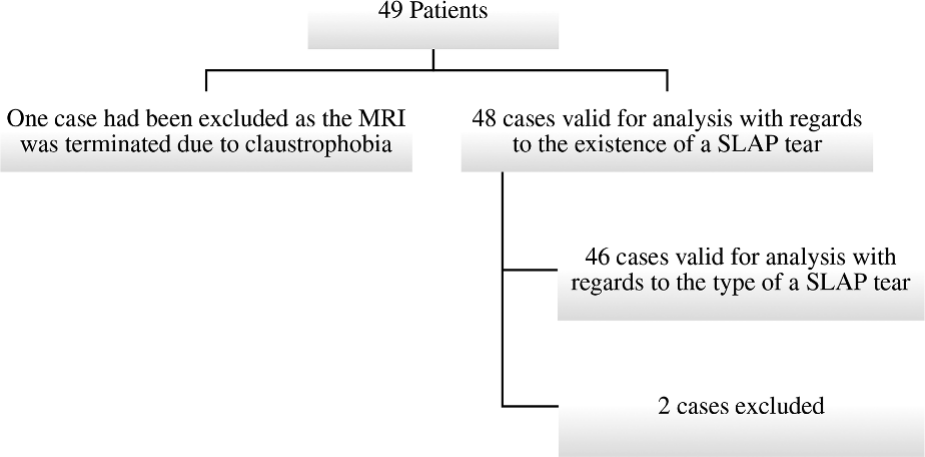


**Figure 3.**
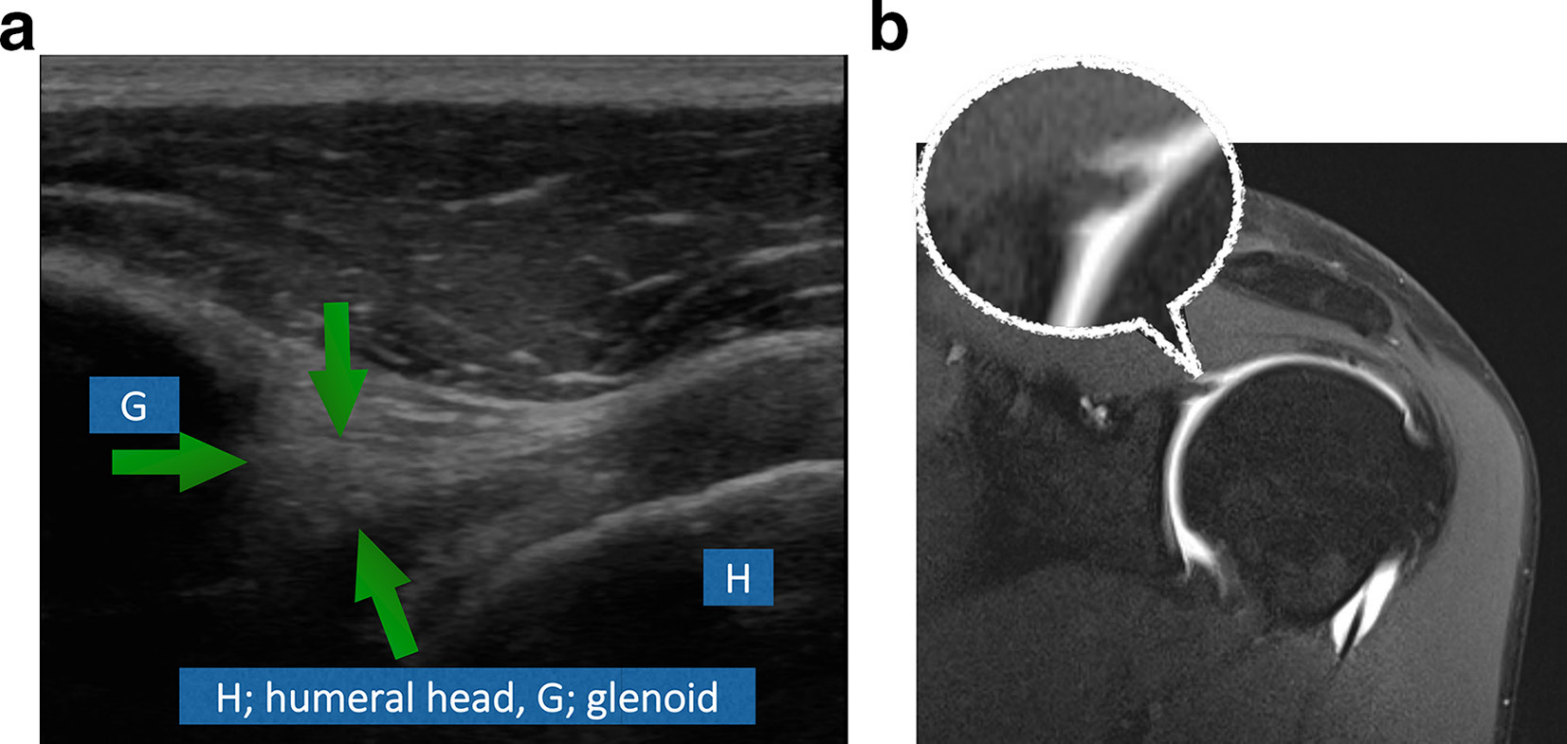
(A) A 40-year-old male patient who came with a history of recurrent subluxation. His MRI shoulder (Figure 3b) shows a normal superior labrum similar to the ultrasound findings (Figure 3A). The superior labrum appears as an echogenic triangular structure on ultrasound.

**Figure 4.**
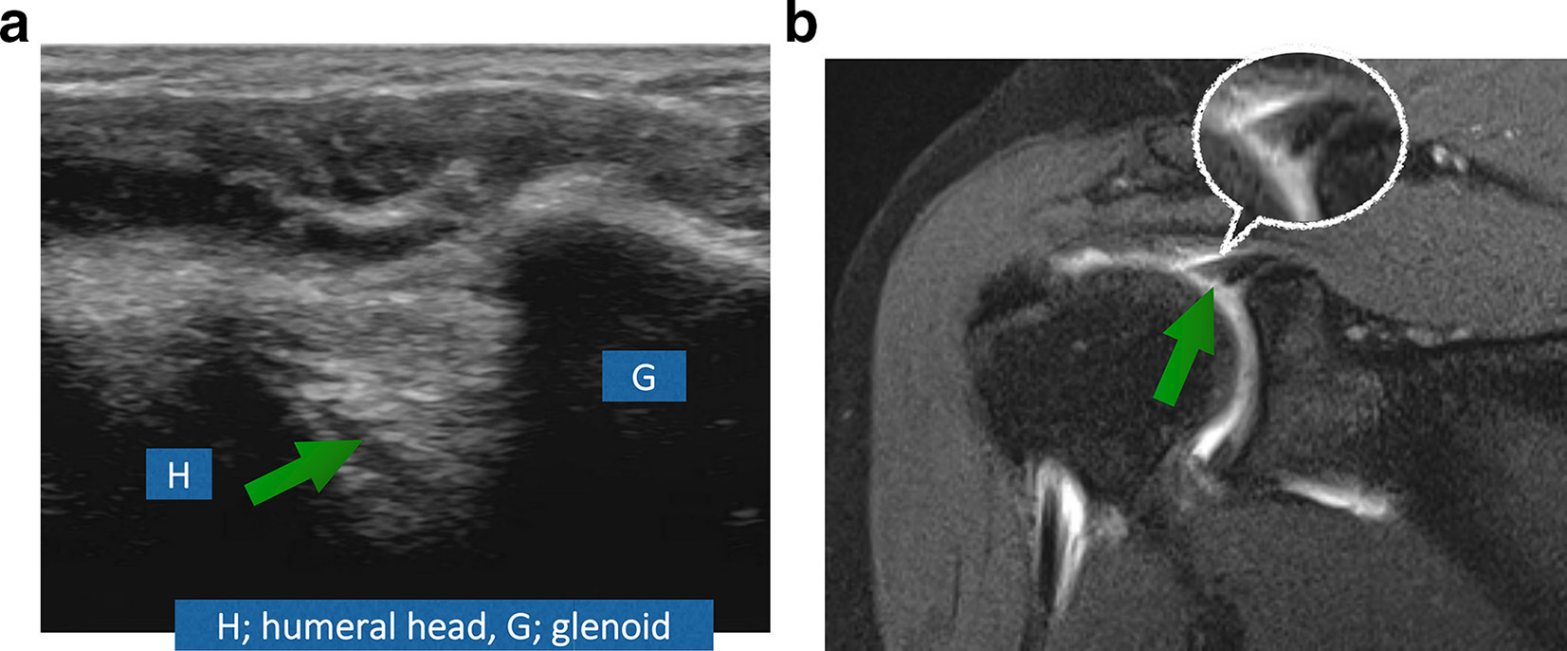
(A) A 22-year-old male patient with recurrent subluxations. Both ultrasound (Figure 4A) and MRI (Figure 4B) show fraying of the labrum in keeping with Type 1 SLAP lesion.

**Figure 5.**
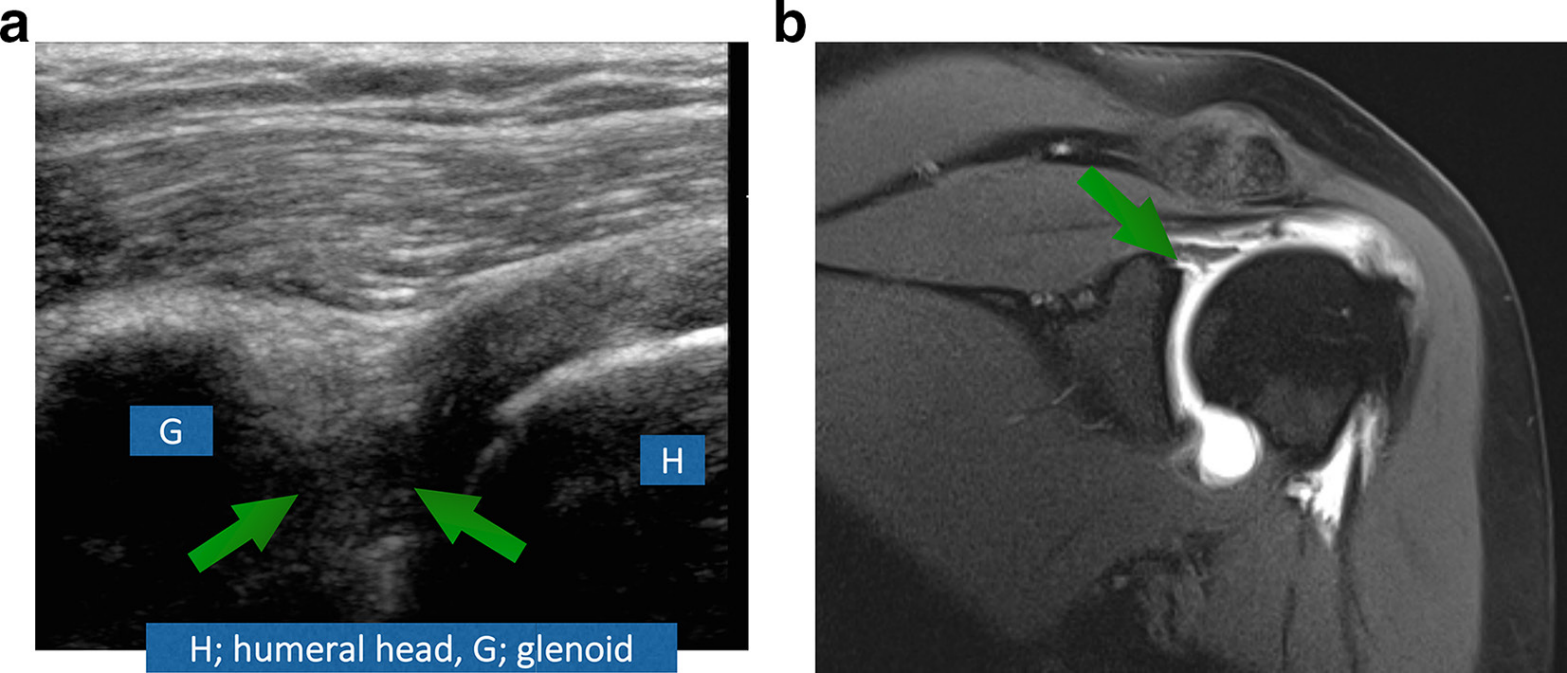
(A) A 37-year-old male patient with history of trauma. Ultrasound (Figure 5A) shows hypoechoic tear (Greenarrow) extending in the labrum parallel to its attachment to the bony glenoid in keeping with SLAP Type 2 lesion. Same findings are seen on MRI (Figure 5B).

**Figure 6.**
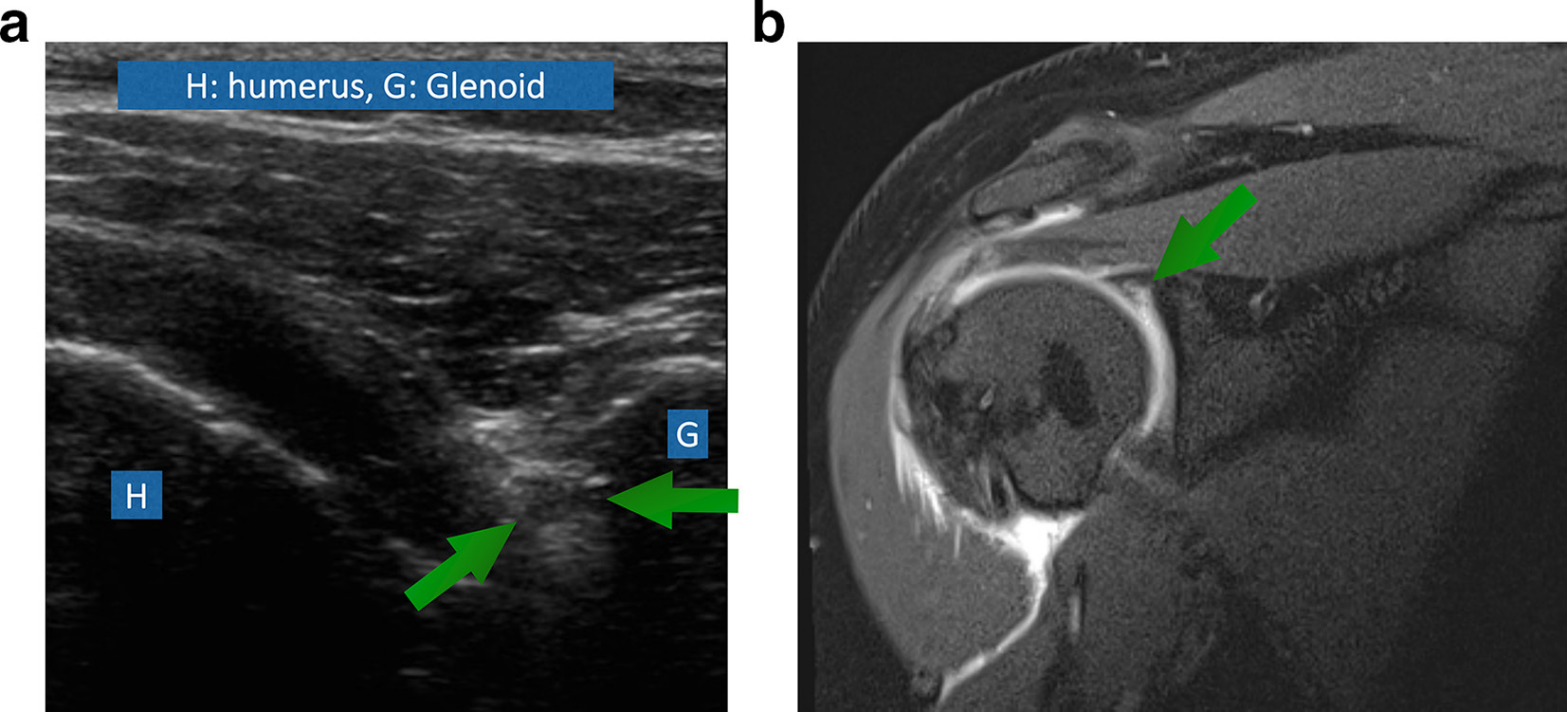
(A) A 38-year-old female patient came with recurrent subluxations. Ultrasound (Figure 6A) shows hypoechoic labral tear extending perpendicularly to its attachment to the bony glenoid in keeping with Type 3 SLAP lesion. Similar findings are seen on MRI (Figure 6B). Arrows depict the tear.

In our study, 48 shoulders were evaluated for the existence of SLAP lesions using both ultrasound and MRI. 19 normal labral cases were correctly identified by ultrasound using MRI as a standard of reference (Figure 3). Five cases with Type 1 SLAP lesions were correctly identified using ultrasound (Figure 4A,B). Similarly, 10 shoulders with Type 2 SLAP lesions were correctly diagnosed by ultrasound (Figure 5A,B). Type 3 SLAP lesion was correctly identified in six cases in our study (Figure 6A,B).

MRI and ultrasound both demonstrated a SLAP tear in 27 of the 48 patients. MRI and ultrasound were in agreement on the absence of a tear in 19 patients. There was discrepancy between ultrasound and MRI in two cases regarding the presence or absence of a tear. MRI showed two tears that were not shown on ultrasound. Overall, the MRI and ultrasound were agreeable on the presence or absence of tear in 95.8% of the patients (*n* = 46/48) (Table 1).

**Table 1.** The existence of SLAP tear on ultrasound and MR arthrogram

			SLAP Tear-ultrasound		Total
			No tear	Yes tear	
SLAP tear-MRI	No tear	Count	19	0	19
		Expected count	8.3	10.7	19.0
	Yes tear	Count	2	27	29
		Expected count	12.7	16.3	29.0
Total		Count	21	27	48
		Expected count	21.0	27.0	48.0

SLAP, superiorlabral anteroposterior.

Regarding the type of SLAP tear, there was agreement in 22 cases (83.7%) and disagreement in 5 (16.3%) (Table 2). A *χ* ^2^ statistic tests the assumption that the frequency of events is evenly distributed. The extent to which the observed frequency of events varies from the expected provides a measure of the relationship.^9^ Technically, the expected count for MRI-0 should be 19 divided by 5 or 3.8 in each of the 5 cells for ultrasound. However, the calculation also takes chance into account and so estimates a more natural distribution that still maintains the assumption that there is no relationship between MRI and ultrasound codes. As we can see from the table, the agreement is much higher than expected by chance.

**Table 2.** The types of SLAP lesions on ultrasound and MR arthrogram

			Type-ultrasound					Total
			0	1	2	3	4	
Type-MRI	**0**	Count	19	0	0	0	0	19
		Expected count	9.1	2.1	4.5	2.9	.4	19.0
	**1**	Count	1	5	1	0	0	7
		Expected count	3.3	.8	1.7	1.1	.2	7.0
	**2**	Count	2	0	10	1	0	13
		Expected count	6.2	1.4	3.1	2.0	.3	13.0
	**3**	Count	0	0	0	6	0	6
		Expected count	2.9	.7	1.4	.9	.1	6.0
	**4**	Count	0	0	0	0	1	1
		Expected count	.5	.1	.2	.2	.0	1.0
Total		Count	22	5	11	7	1	46
		Expected count	22.0	5.0	11.0	7.0	1.0	46.0

SLAP, superior labral anteroposterior.

MRI and ultrasound findings for the presence or absence of a tear and for grading of the tears were assessed with κ statistics, as this is recognized as widely accepted measure of agreement. The two modalities demonstrated substantial agreement on the presence or absence of tear (κ = 91.4 %, *p* < 0.001) as well as the grading of the tear (κ = 84.4 %, *p* < 0.001). As for all statistical tests, α was set to 0.05, which indicates the highest acceptable probability that any relationship between the data occurred by chance. A *p* value < 0.05 indicates that there is less than a 5% chance that the observed relationship between the data occurred by chance. Given the large differences between the observed and the expected frequencies, there is a very small likelihood, (*p* < 0.001) that the differences occurred entirely by chance.

## Discussion

During the last two decades, several studies demonstrated that the glenoid labrum could be seen and evaluated by ultrasound. In 1998, Schydlowsky et al had concluded that ultrasound can be used to identify the anterior labrum and distinguish between degenerate and normal labrum.^10^ In 2000, Taljanovic et al published an article to estimate the utility of sonography of glenoid labrum with cadaveric arthroscopic correlation.^11^ In their study, ultrasound has a sensitivity of 67% with regards to differentiating labral lesions from other conditions including normal cases as well as cases with labral degeneration. In this study, sonography of inflexible cadavers was a limitation and could have contributed to the incomplete assessment of superior labrum.^11^ Additionally, few labral tears were seen (3/80) and the grade of SLAP lesions was not assessed. Accurate grading of the SLAP tear is essential as the grading changes the treatment plan. Generally, the treatment of Type 1 SLAP lesions in many cases is non-surgical. The other SLAP lesions particularly Type 2 require arthroscopic repair specially in young and active patients.^12^

In this pilot study, the feasibility of high resolution ultrasound for SLAP tears was assessed and compared with MR arthrogram, to evaluate its accuracy. The two modalities show substantial agreement on the existence of SLAP tears. This fact supports the use of high resolution ultrasound as a screening tool, given the advantage of it being fast and readily available. Its use would definitely shorten the long waiting times for MRI/MR arthrogram especially by excluding those cases which do not demonstrate a tear on high resolution ultrasound. The two modalities show a lesser degree of agreement with regards to the grading of the tear (κ = 84.4%) which is still excellent. The variation in accuracy between the two modalities is expected as the inter observer agreement for SLAP tears utilizing MRI as the sole modality is found to be substantial in the literature (κ = 0.77) to moderate (κ = 0.52).^13^

There are multiple superior labral normal variants which challenge interpretation of shoulder MRI specially the presence of sublabral recess which could be seen in up to 2.46% of the cases.^14^ Despite the high accuracy of MRI, the discrimination between sublabral recess and SLAP lesions remains difficult. The differentiation between pathological conditions and normal variants is crucial to the referring physician as this will the change the management dramatically. We did not study these normal variants in our ultrasound cases but this is worth considering in future studies.

Ultrasound has several advantages when compared to MRI including portability, accessibility, rapidity and lower costs.^15^ These advantages support its use as a screening tool. Additionally, ultrasound can be used as an alternative to MRI, when there are contraindications to performing MRI. The incidence of claustrophobia and prematurely terminated examination due to claustrophobia is not negligible and is estimated as 1.97% and 1.22% respectively.^16^ Ultrasound is obviously more patient friendly when it comes to claustrophobia.

## Limitations

There are some limitations to our study particularly the small sample size as it is a pilot study. 48 cases were evaluated over a 2-year period. A study with a larger sample would be more representative. As mentioned earlier, large body habitus causing inadequate penetration of the ultrasound beam was a limitation of this technique, similar to the use of ultrasound elsewhere in the body. Another limitation is that only one fellowship trained MSK radiologist performed and interpreted the ultrasound and MRI cases, separately. Having at least two MSK radiologists perform and interpret all the cases would help evaluate interobserver reliability as well. Finally, the accuracy of both ultrasound and MRI could not be accurately assessed as only few patients underwent arthroscopic evaluation which is the gold standard for diagnosis of SLAP tears.

## Future directions

High resolution ultrasound of superior labral tears shows good promise for diagnosis of superior labral tears, comparable to MR arthrogram. It can be used as a screening tool, given its accessibility, portability, rapidity and low cost. However, it is necessary to be familiar with the relevant ultrasound anatomy & technique and therefore involves a learning curve. A study with a larger sample with surgical correlation is recommended in the future.

## Conclusions

The feasibility of high resolution ultrasound for SLAP tears was assessed and compared to MR arthrogram in this pilot study. MR arthrogram and ultrasound demonstrated substantial agreement on the presence or absence of SLAP tears and the grading of the tears. Therefore, this pilot study supports the use of high resolution ultrasound as a screening tool for SLAP tears, especially as it is readily available, fast and inexpensive.
